# Differential and Cooperative Cell Adhesion Regulates Cellular Pattern in Sensory Epithelia

**DOI:** 10.3389/fcell.2016.00104

**Published:** 2016-09-15

**Authors:** Hideru Togashi

**Affiliations:** Division of Molecular and Cellular Biology, Department of Biochemistry and Molecular Biology, Kobe University Graduate School of MedicineKobe, Japan

**Keywords:** cell adhesion molecules, cadherins, nectins, sensory organs, cell sorting, mosaic cellular pattern, self-organization

## Abstract

Animal tissues are composed of multiple cell types arranged in complex and elaborate patterns. In sensory epithelia, including the auditory epithelium and olfactory epithelium, different types of cells are arranged in unique mosaic patterns. These mosaic patterns are evolutionarily conserved, and are thought to be important for hearing and olfaction. Recent progress has provided accumulating evidence that the cellular pattern formation in epithelia involves cell rearrangements, movements, and shape changes. These morphogenetic processes are largely mediated by intercellular adhesion systems. Differential adhesion and cortical tension have been proposed to promote cell rearrangements. Many different types of cells in tissues express various types of cell adhesion molecules. Although cooperative mechanisms between multiple adhesive systems are likely to contribute to the production of complex cell patterns, our current understanding of the cooperative roles between multiple adhesion systems is insufficient to entirely explain the complex mechanisms underlying cellular patterning. Recent studies have revealed that nectins, in cooperation with cadherins, are crucial for the mosaic cellular patterning in sensory organs. The nectin and cadherin systems are interacted with one another, and these interactions provide cells with differential adhesive affinities for complex cellular pattern formations in sensory epithelia, which cannot be achieved by a single mechanism.

## Introduction

Vertebrates possess highly developed sense organs such as the eyes, ears, nose, and tongue. These sense organs detect information about different environments and convert extracellular stimuli into electrical signals. These signals are mediated by specialized sensory epithelia. For example, in vertebrates, the perception of sound is mediated by the auditory epithelium located in the cochlea of the inner ear, and the perception of smell is mediated by the olfactory epithelium inside the nasal cavity. These sensory epithelia are typically composed of sensory cells and non-sensory supporting cells. Interestingly, the same types of sensory cells in the sensory epithelia are separated from one another to form alternating mosaic patterns. These mosaic patterns observed in the sensory organs are evolutionarily conserved among a wide range of species, and are thought to be important for the sensory functions. During the development of the auditory epithelium, sensory hair cells and supporting cells are thought to be segregated through the process of lateral inhibition mediated by Notch–Delta signaling, and such processes themselves might contribute to the spatial separation of these cells (Zhang et al., 2000; Zine et al., 2001). Expression of Notch 1 and its ligand Jagged 2 is restricted to supporting cells and hair cells, respectively, in the auditory epithelium. In a previous study, genetic inactivation of Notch signaling did not impair the cellular pattern, but did result in an increased number of hair cells (Lanford et al., 1999). These observations suggest that lateral inhibition alone is insufficient to create the checkerboard-like cellular pattern. Recent progress has suggested that cellular rearrangements could also play a role in the mosaic pattern formation in the auditory epithelium. The question then arises as to how cell adhesion molecules regulate the complex and elaborate cellular patterns in the sensory organs. In this review, recent advances in cellular patterning within the sensory epithelia are introduced and discussed.

## Cell–cell adhesion and cellular patterning

Cell–cell adhesion systems are involved in many aspects of morphogenesis during development, including not only adhesion, but also movement, proliferation, survival, differentiation, and polarization. Various morphogenetic changes in tissues in developing embryos can be brought about by local cell rearrangements. Differential adhesion and cortical tension have been proposed to promote cell rearrangements and cell sorting in cell aggregates (Foty and Steinberg, 2005; Steinberg, 2007). Steinberg's theory of differential cell adhesion explains cell sorting as follows: when cell adhesion between cells of the same type is stronger than that between cells of different types, the two types of cells should become segregated; conversely, when cell adhesion is stronger between cells of different types, the two types of cells should become intermingled with one another. Although this differential cell adhesion theory suggests roles for self-organized cell movements in various epithelia, it remains unclear how this strategy for cell sorting is used during morphogenesis *in vivo*. Differential mechanical tension also plays an important role in cellular rearrangements (Lecuit, 2005; Heisenberg and Bellaiche, 2013). For example, during *Drosophila* germband extension, junctions are remodeled through the polarized recruitment of myosin II within the epithelium (Bertet et al., 2004). The contractile activity of myosin II creates local tension that orients the disassembly of E-cadherin junctions. In the case of neural-tube closure, polarized constriction of neuroepithelial adherens junctions (AJs) induces the convergence of their apical domains toward the midline of the neural plate (Nishimura et al., 2012). These observations suggest that anisotropic extensions and contractions of cell–cell junctions are used for cellular rearrangements in various epithelia.

## Roles of cadherins and nectins in cellular patterning

**Figure 1:**
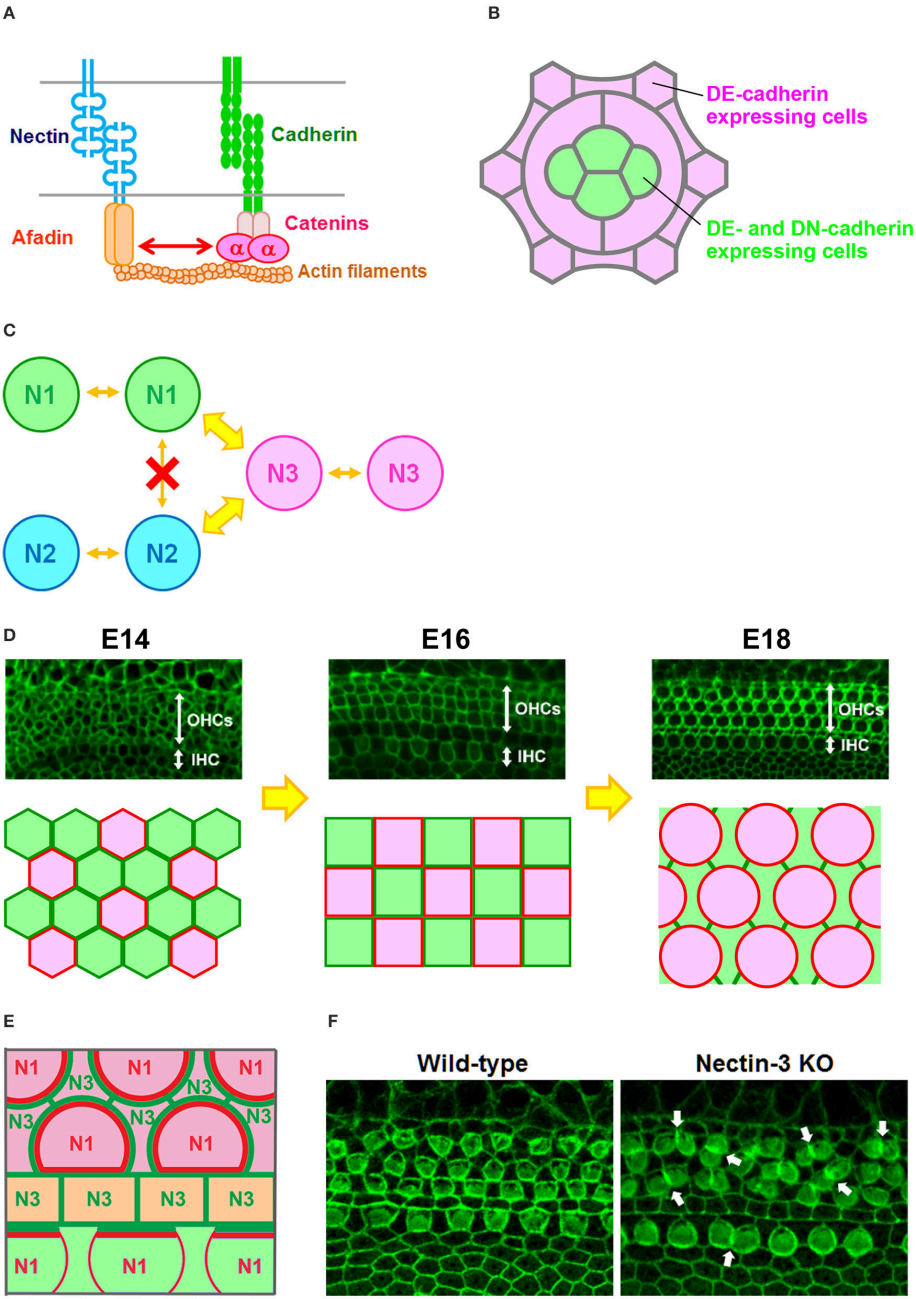
**Checkerboard-like cellular pattern in the mouse auditory epithelium. (A)** Molecular interactions between nectins and cadherins (Takai and Nakanishi, 2003). α, α-catenin. **(B)** Expression pattern of DE-cadherin and DN-cadherin in the *Drosophila* retina (Hayashi and Carthew, 2004). All cells express DE-cadherin, but only cone cells express DN-cadherin. **(C)** Homophilic and heterophilic trans-interactions between nectins (Takai and Nakanishi, 2003). N1, nectin-1; N2, nectin-2; N3, nectin-3. Wide arrows, strong interactions; narrow arrows, weak interactions. **(D)** Cellular rearrangement of the auditory epithelium from embryonic day (E) 14 to E18 (McKenzie et al., 2004; Togashi et al., 2011). (Upper) Localization of ZO-1 at the apical surface of the auditory epithelium. OHCs, outer hair cells; IHC, inner hair cell. (Lower) Schematic illustrations of the cellular rearrangements. Red, differentiated hair cells; green, supporting cells. **(E)** Expression pattern of nectins and cadherins in the auditory epithelium (Simonneau et al., 2003; Togashi et al., 2011). Nectin-1 is expressed in hair cells, while nectin-3 is expressed in supporting cells. E-cadherin is expressed in the region including the outer hair cells (pink), and N-cadherin is expressed in the medial inner hair cell region (light green). Pillar cells express P-cadherin (light yellow). N1, nectin-1; N3, nectin-3. Red or green lines indicate nectin protein localizations at cell–cell boundaries. **(F)** Cellular patterning in the auditory epithelia of wild-type and nectin-3 knockout (KO) mice (Togashi et al., 2011). Arrows point to examples of aberrantly attached hair cells.

The major cell adhesion molecules at AJs are cadherins and nectins (Figure 1A; Takai et al., 2008; Meng and Takeichi, 2009). Cadherins are essential for maintaining multicellular structures, and play a role in vital processes such as embryogenesis, pattern formation, and maintenance of specific tissue architectures. Cadherins are Ca^2+^-dependent cell–cell adhesion molecules that constitute a superfamily, and are grouped into subfamilies designated classic cadherins and proto-cadherins. Here, for convenience, classic cadherins are simply referred to as cadherins. Cadherin molecules associate with p120 catenin and β-catenin via their cytoplasmic domain, and β-catenin in turn binds to α-catenin. α-Catenin can bind to F-actin, an interaction thought to be crucial for cadherins to create firm cell adhesions (Meng and Takeichi, 2009). The major role of cadherins is to connect cells expressing the same cadherins through homophilic interactions. Through these properties of cadherins, cells in mixed cultures of cell lines expressing E- or N-cadherin were observed to form separate aggregates (Nose et al., 1988; Katsamba et al., 2009), while differential levels of cadherin expression in two transfected cell populations caused one cell population to segregate internally or externally from the other cell population (Friedlander et al., 1989; Steinberg and Takeichi, 1994). The multicellular hexagonal lattice formation of the *Drosophila* retina is thought to arise through a cell-sorting process (Tepass and Harris, 2007). All cells in the retina express DE-cadherin, whereas only the cone cells express DN-cadherin (Figure 1B). The cone cell shape is formed by differential cadherin-mediated adhesion (Hayashi and Carthew, 2004). Differential expression of DN-cadherin within cone cells causes these cells to form an overall shape that minimizes their surface contact with surrounding cells. These observations indicated that simple patterned expression of cadherin results in a complex spatial pattern of cells in the visual system of *Drosophila*.

Nectins comprise a family of immunoglobulin-like molecules with four members, nectin-1, -2, -3, and -4. The most important property of this family is that nectins interact with other nectins in homophilic or heterophilic manners. Furthermore, nectins prefer heterotypic partners to homotypic partners, and their heterophilic interactions produce stronger cell–cell adhesions than their homophilic interactions (Figure 1C; Fabre et al., 2002; Yasumi et al., 2003; Martinez-Rico et al., 2005; Harrison et al., 2012). Owing to these properties of nectins, cells in mixed cultures of cell lines expressing different nectins became arranged in a mosaic pattern (Togashi et al., 2006, 2011). Several studies have shown that heterophilic interactions of nectins are present in cell–cell adhesions between Sertoli cells and germ cells in the testis (Ozaki-Kuroda et al., 2002; Mueller et al., 2003; Inagaki et al., 2005), between commissural axons and floor plate cells in the neural tube (Okabe et al., 2004), between pigment cell and non-pigment cell layers of the ciliary epithelium in the eye (Inagaki et al., 2005), between ameloblasts and stratum intermedium cells in the developing tooth (Yoshida et al., 2010), and between dendrites and axons of hippocampal neurons during synaptogenesis (Honda et al., 2006; Togashi et al., 2006). These observations suggest that heterophilic interactions of nectins between different cell types are involved in many aspects of organogenesis during development.

The cadherin and nectin systems are associated during cell–cell junction formation through their intercellular interactions (Figure 1A). It has been suggested that nectins and afadin initially form cell–cell adhesions and then recruit cadherins to the nectin-based cell–cell adhesion sites to accelerate the formation of cadherin-dependent junctions (Tachibana et al., 2000; Honda et al., 2003). However, it remains unclear which of these molecules results in the recruitment of nectin and cadherin into AJs. It has been reported that cadherins control nectin recruitment into AJs through actin clustering (Troyanovsky et al., 2015). It is possible that both actin bundle formation and adhesion complex clustering mutually regulate cell–cell junction formation. Although both afadin and α-catenin are known to be essential for the associations of nectins and cadherins, the underlying mechanism for how the nectin–afadin system is interacted with the cadherin–catenin system remains unknown (Pokutta and Weis, 2000; Tachibana et al., 2000; Takai and Nakanishi, 2003; Takai et al., 2008).

## Checkerboard-like pattern formation in the auditory epithelium

The auditory epithelium of the mammalian inner ear consists of inner hair cells, outer hair cells, and at least four different types of supporting cells. The hair cells and supporting cells are organized in a checkerboard-like pattern, such that each hair cell is separated from another hair cell by a supporting cell, forming an alternating mosaic. Cellular rearrangements are a possible mechanism for the patterning in the inner ear. In the developing auditory epithelium, the hair cells and supporting cells continue to change their position and alignment beyond the period of terminal mitoses for cells (Figure 1D; Chen et al., 2002; McKenzie et al., 2004). However, the driving force for the cellular rearrangements required for the checkerboard-like patterning is not well understood. Previous studies showed that E- and N-cadherins have complementary expression patterns within the auditory epithelium (Whitlon, 1993; Simonneau et al., 2003; Chacon-Heszele et al., 2012), with E-cadherin detected in the region including the outer hair cells, and N-cadherin restricted to cells in the medial inner hair cell region. Thus, the homophilic adhesive property of cadherins alone cannot explain the mosaic cellular patterning. A mathematical model suggested that the checkerboard-like pattern could be generated by a mixture of two cell types, when their heterotypic cell–cell adhesions dominate over their homotypic cell–cell adhesions (Honda et al., 1986). Our previous study showed that the heterophilic interactions of different nectins regulate the checkerboard-like cellular patterning in the mouse auditory epithelium (Togashi et al., 2011). In the mouse cochlea from an early developmental stage, nectin-1 and nectin-3 are complementarily expressed in hair cells and supporting cells, respectively, and become condensed at heterophilic junctions (Figure 1E). Molecular interactions occur between nectin-1 on hair cells and nectin-3 on supporting cells, and the majority of these molecules are recruited to heterophilic binding sites, such that these biased cell–cell adhesions contribute to the checkerboard-like pattern formation. Genetic deletion of nectin-1 or nectin-3 causes the redistribution of their heterophilic partners to ectopic sites and induces aberrant attachments between hair cells, resulting in disruption of the checkerboard-like pattern (Figure 1F). These observations indicated that nectins play a key role in the formation of the checkerboard-like pattern of hair cells and supporting cells in the auditory epithelium.

## Mosaic pattern formation in the olfactory epithelium

**Figure 2:**
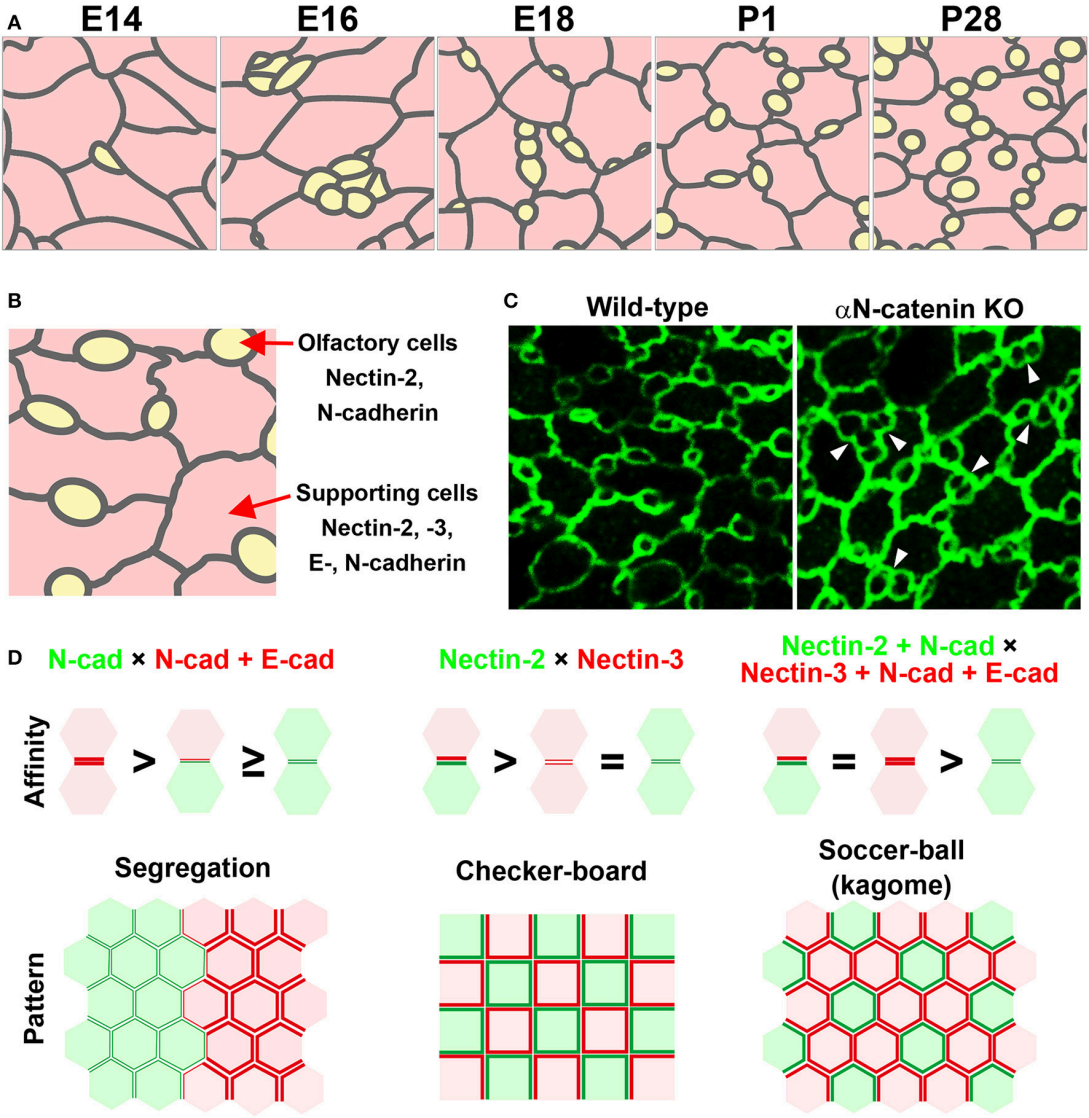
**Mosaic cellular pattern in the mouse olfactory epithelium. (A)** Schematic illustrations of the apical surface of the mouse olfactory epithelium from embryonic day (E) 14 to postnatal day (P) 28 (Katsunuma et al., 2016). Yellow, olfactory cells; pink, supporting cells. **(B)** Expression pattern of nectins and cadherins in the olfactory epithelium (Katsunuma et al., 2016). **(C)** Cellular patterning in the olfactory epithelia of wild-type and αN-catenin knockout (KO) mice (Katsunuma et al., 2016). Arrowheads point to examples of aberrantly attached olfactory cells. **(D)** Cellular patterns generated by various combinations of cell adhesive affinities (Katsunuma et al., 2016). (Upper) Schematic illustrations of the relative adhesive affinity between the cells. (Lower) Schematic illustrations of the generated cellular patterns.

The olfactory epithelium, which is located inside the nasal cavity in mammals, is a specialized sensory epithelium involved in odor perception. The olfactory epithelium is a pseudostratified columnar epithelium composed of olfactory cells, supporting cells, and basal cells. These cells are stereotypically layered from the apical to basal side in the olfactory epithelium. When the luminal surface of the olfactory epithelium is observed from the apical side, ciliated olfactory cells and several types of supporting cells are arranged in a specialized mosaic pattern (Figure 2A; Cuschieri and Bannister, 1975; Steinke et al., 2008). The most characteristic aspect of this cellular pattern is that small round olfactory cells are interspersed between hexagonal supporting cells. How is this pattern established? During development, the olfactory cells and supporting cells dynamically arrange themselves to form a mosaic pattern (Figure 2A; Katsunuma et al., 2016). The immature olfactory cells are initially clustered at the boundary between neighboring supporting cells at an early development stage. Subsequently, the clustered olfactory cells gradually become separated from one another, and each olfactory cell becomes fully surrounded by supporting cells. These observations imply that cellular rearrangements are required for the mosaic patterning in the olfactory epithelium. However, the cellular rearrangements in the developing olfactory epithelium are unlike those in the developing inner ear. As the initial pattern shows segregation of the olfactory cells from the supporting cells, lateral inhibition is not likely to be involved in the mosaic pattern formation. Interestingly, the olfactory cells and supporting cells express different cadherins and nectins (Steinke et al., 2008; Katsunuma et al., 2016). Specifically, the supporting cells express E-cadherin, N-cadherin, nectin-2, and nectin-3, while the olfactory cells express N-cadherin and nectin-2 (Figure 2B). What mechanism coordinates the nectin and cadherin adhesion systems in the mosaic cellular patterning? Our recent study showed cooperative actions of nectins and cadherins in the mosaic cellular patterning within the mouse olfactory epithelium. Nectin-2 on olfactory cells interacts with nectin-3 on supporting cells, and this trans-heterophilic interaction promotes homophilic trans-interactions of N-cadherins between the olfactory cells and the supporting cells. The trans-interactions of cadherins at the heterotypic O-S (olfactory cell–supporting cell) boundary are stronger than those at the homotypic O-O (olfactory cell–olfactory cell) boundary, resulting in separation of the olfactory cells. The adhesiveness of the O-S boundary is as strong as that of the S-S (supporting cell–supporting cell) boundary, because E-cadherin exclusively accumulates in the homophilic S-S junctions. However, the adhesiveness of the O-O boundary is not sufficient to sustain these contacts, resulting in separation of the olfactory cells. Collectively, these findings indicate the involvement of heterophilic binding between nectin-2 on olfactory cells and nectin-3 on supporting cells as well as the selective recruitment of E-cadherin to homophilic interactions between nectin-3-expressing supporting cells. The association of nectins with α-catenin through cytoplasmic interactions is necessary for the efficient clustering of cadherin molecules at cell–cell adhesion sites, and for enhancing the adhesion activity of the clustered cadherin molecules. Genetic deletion of αN-catenin, a subtype of α-catenin that is specifically expressed in the olfactory cells in the olfactory epithelium, causes aberrantly attached of the olfactory cells each other, resulting in disruption of the cellular pattern (Figure 2C). The data implicate that intra-cellular mechanisms implicate in the regulation of the activity of cadherins. Mathematical modeling supports the idea that the strength of adhesion between olfactory cells and supporting cells is greater than that between olfactory cells and equivalent to that between supporting cells, resulting in cellular intercalation of the supporting cells between the olfactory cell junctions. These observations demonstrate that the cooperative action of nectins and cadherins leads to the intercalation of supporting cells between the olfactory cells, resulting in olfactory cell dispersion in the olfactory epithelium, and also suggest that combinatorial expression of nectins and cadherins contributes to the production of the complex cell patterns of sensory organs, which cannot be achieved by a single mechanism (Figure 2D).

## Concluding remarks

Herein, the roles of the cell–cell adhesion molecules nectins and cadherins in the unique cellular pattern formation of sensory organs have been introduced and discussed. Many different types of cells in tissues express various types of cell adhesion molecules. As multiple adhesion systems appear to cooperate at the organogenesis level, it will be of great interest to determine whether they act in parallel or hierarchical manners. As mentioned above, the nectin-dependent differential distribution of cadherins leads to extension or shrinkage of cell–cell junctions, thereby contributing to the cell intercalations and mosaic patterning. However, cooperative mechanisms between nectins, cadherins, and cortical tension are likely to contribute to the production of the complex cell patterns, and our current understanding of the cooperative roles between these mechanisms is insufficient to explain the complex mechanisms underlying cellular patterning. Elucidating the combined mechanisms between differential adhesion and actin–myosin contractility will provide a more detailed picture of cellular patterning. How does the cooperative action of nectins and cadherins regulate actin–myosin contraction and generate differential adhesion? Cell cortical tension is caused by actin–myosin contraction and cell–cell adhesion, and actin cytoskeletal anchoring of cell adhesion molecules is achieved by various actin-binding proteins. Previous studies have shown that mechanical stress on cadherin adhesion complexes modifies their cytoskeletal anchoring strength (Yonemura et al., 2010), and that cadherin oligomerization stiffens these molecules anchored to the actin cytoskeleton (Strale et al., 2015). To understand the mechanism for the production of the complex cell patterns and structures in epithelia, deciphering the intracellular and intercellular signaling mechanisms will provide insights into how the cooperation of cell–cell adhesion and cortical tension contributes to self-organization of cells into tissues.

## Author contributions

The author confirms being the sole contributor of this work and approved it for publication.

## Funding

The author's laboratory was supported by KAKENHI Grant Numbers 25111716, 25127710, 25440107, and 26400205, and by a grant from the Takeda Science Foundation.

### Conflict of interest statement

The author declares that the research was conducted in the absence of any commercial or financial relationships that could be construed as a potential conflict of interest.
